# The Effect of Protease Inhibitors on the Induction of Osteoarthritis-Related Biomarkers in Bovine Full-Depth Cartilage Explants

**DOI:** 10.1371/journal.pone.0122700

**Published:** 2015-04-24

**Authors:** Yi He, Qinlong Zheng, MengMeng Jiang, Shu Sun, Thorbjørn G. Christiansen, Moustapha Kassem, Morten A. Karsdal, Anne C. Bay-Jensen

**Affiliations:** 1 NordicBioscience, Herlev, Danmark; 2 NordicBioscience China, Beijing, P.R. China; 3 Orthopedic Department, Gentofte University Hospital, Hellerup, Denmark; 4 Molecular Endocrinology Laboratory (KMEB), Odense University Hospital, Odense, Denmark; INRS, CANADA

## Abstract

**Objective:**

The specific degradation of type II collagen and aggrecan by matrix metalloproteinase (MMP)-9, -13 and ADAMTS-4 and -5 (aggrecanase-1 and -2) in the cartilage matrix is a critical step in pathology of osteoarthritis (OA). The aims of this study were: i) To investigate the relative contribution of ADAMTS-4 and ADAMTS-5 to cartilage degradation upon catabolic stimulation; ii) To investigate the effect of regulating the activities of key enzymes by mean of broad-spectrum inhibitors.

**Methods:**

Bovine full-depth cartilage explants stimulated with tumor necrosis factor alpha (TNF-α) and Oncostatin M (OSM) were cultured for 21 days with or without a number of inhibitors targeting different types of proteases. Monoclonal antibodies were raised against the active sites of ADAMTS-4, -5, MMP-9 and -13, and 4 ELISAs were developed and technically validated. In addition, the established AGNxI (ADAMTS-degraded aggrecan), AGNxII (MMP-degraded aggrecan), and CTX-II (MMP-derived type II collagen) were quantified in the explants-conditioned media.

**Results:**

We found that: i) Active ADAMTS-4, MMP-9, -13 were released in the late stage of TNF-α/ OSM stimulation, whereas no significant active ADAMTS-5 was detected in either extracts or supernatants; ii) Active ADAMTS-4 was primarily responsible for E^373^-^374^A bond cleavage in aggrecan in this setting; and iii) The compensatory mechanism could be triggered following the blockage of the enzyme caused by inhibitors.

**Conclusions:**

ADAMTS-4 appeared to be the major protease for the generation of ^374^ARGS aggrecan fragment in the TNF-α/OSM stimulated bovine cartilage explants. This study addresses the need to determine the roles of ADAMTS-4 and ADAMTS-5 in human articular degradation in OA and hence identify the attractive target for slowing down human cartilage breakdown.

## Introduction

Osteoarthritis (OA) is the most common joint disease. The high prevalence and in many cases severe disability make OA a huge economical burden for societies [1]. A hallmark of OA is the progressive degradation of the articular cartilage. This suggests that the balance between anabolic and catabolic processes has been disrupted [2].

Aggrecan, a large aggregating proteoglycan, and type II collagen are the most abundant constituents of the cartilage matrix. Increased proteolytic processing of aggrecan and type II collagen is related to the pathology of OA in human. A disintegrin and metalloproteinase with thrombospondin motifs-4 (ADAMTS-4, or aggrecanase 1) and -5 (ADAMTS-5, or aggrecanase 2) are considered to be responsible for the deleterious cleavage at E^373^↓^374^A within the inter-globular domain (IGD) of aggrecan, although this cleavage site is not the preferred action of any of the two enzymes [3,4]. It is still debated which of the two aggrecanases is mainly responsible for the aggrecan degradation during human OA. Another important cleavage between N^341^↓^342^F within IGD is attributed to the action of MMPs aggrecanolysis [5].

Fibrillar collagens, including type II collagen, are highly stable molecules and only sensitive to cleavage by few enzymes. MMP-1 and MMP-13 are both able to degrade type II collagen, whereas MMP-13 is the most efficient of the mentioned MMPs for this purpose [6]. Over-expression of MMP-13 in transgenic mice induced spontaneous cartilage degradation [7]. In addition, a high expression of MMP-13 has been measured in patients with cartilage destruction [8]. These findings suggest that MMP-13 plays a critical role in the degradation of cartilage matrix in OA. However, the expression of MMP-9 (also known as gelatinase B) is enhanced in OA cartilage suggesting that it might take part in the cartilage destruction as well [9].

A combination of tumor necrosis factor (TNF)–α and Oncostatin M (OSM) added to bovine cartilage explants can mimic the cartilage degradation occurring in OA driven by pro-inflammatory factors [10,11]. The enzymatic processes induced by TNF-α/OSM have demonstrated that high level of aggrecanase-mediated aggrecan degradation happens prior to the induction of MMP-mediated aggrecan and type II collagen degradations [12,13]. The late release of MMP-derived cartilage degradation products could be partly explained by the requirement of proteolytic activation before MMPs can be secreted as latent zymogens require proteolytic activation. Thus, the activation of inactive MMPs might be a key step in cartilage degradation [14].

The aims of this study were: i) To investigate the relative contribution of ADAMTS-4 and ADAMTS-5 to bovine cartilage degradation upon catabolic stimulation; and ii) To investigate the effects of regulating the activities of key enzymes during cartilage breakdown by broad-spectrum inhibitors, which may provide important implications for OA therapeutic strategies. We applied and cultured a bovine full-depth cartilage *ex vivo* model stimulated with TNF-α/OSM for 21 days with or without a number of inhibitors targeting different types of proteases. We collected the supernatants of different treatments from nine time points. To measure the levels of active proteases, monoclonal antibodies were raised against the N-terminal sequence of active ADAMTS-4, -5, MMP-9 (unpublished) and -13, and 4 ELISAs were developed and technically validated. These, as well as AGNxI (fragments of ADAMTS-degraded aggrecan), AGNxII (fragments of MMP-degraded aggrecan), and CTX-II (commercial assay, MMP-derived type II collagen fragments) were quantified in the explants-conditioned medium.

## Materials and Methods

All chemicals were obtained from either Sigma-Aldrich (Copenhagen, Denmark) or Merck Millipore (Hellerup, Denmark) unless other stated. Recombinant human active ADAMTS-5 (cat#, 2198AD), human pro-MMP13 (cat#, 511-MM-010), human TNF-α (cat#, 210-TA) were purchased from R&D systems (Abingdon, UK). Dulbecco’s Modified Eagle Medium, Nutrient Mixture F-12 (DMEM: F12) was purchased from Life Technologies (Naerum, Denmark). AlamarBlue was from VWR international (Herlev, Copenhagen). Oncostatin M human (cat#, O9635) was from Sigma-Aldrich (Copenhagen, Denmark). GM6001 (cat#, M5939, Sigma-Aldrich) stock solution was dissolved in DMSO and then 1000x diluted in culture medium to a final concentration of 10μM. Proprotein Convertase (PC) inhibitor (cat#, 537076, Millipore) stock solution was prepared in DMSO and 1000x diluted in culture medium to a final concentration of 10μM. E64 (cat#, E3132, Sigma-Aldrich) was dissolved in sterile water to a final concentration of 10μM.

The streptavidin pre-coated ELISA plates were purchased from Roche (Mannheum, Germany). All ELISA plates were analyzed with the reader from Molecular Devices, SpectraMax M (CA, USA). Active ADAMTS-4, -5, MMP-9, -13, AGNxI and AGNxII lab scale kits were produced in NordicBioscience (Herlev, Denmark). The pre-clinical Cartilage-Laps ELISA was obtained from IDS Ltd. (Boldon, UK).

### Bovine full-depth cartilage (FDC) explants

Bovine cartilage explants were harvested from the proximal femoral condyle of stifle joint of one one-year-old Hereford bull from local butchery. No animal was killed for the purposes of this study. Full-depth cartilage cylinders (3-mm diameter) including superficial zone, middle zone, deep zone and calcified cartilage zone were taken using a biopsy punch (Miltex, Germany) as previously described[18]. The biopsies (~17 mg/each) were washed three times in PBS, and cultured for two days in 96-well plates in serum-free DMEM: F12 media with 1% penicillin and streptomycin added. The AlamarBlue assay and Safranin O/Fast green staining were performed to show that the bovine cartilage explants were sustainable over long-term culture in serum-free medium (S1 and S2 Figs). Biopsies were divided into 5 groups with 8 replicates in each. The groups were as follows: 1). media without (WO) treatment or 2). Media with catabolic agents (T+O media) consisting of TNF-α (20ng/ml) plus OSM (10ng/ml); 3). T+O media + GM6001 (a broad spectrum inhibitor of metalloproteinase); 4). T+O media + E64 (an inhibitor of cysteine protease); 5). T+O media + Furin-like pro-peptide convertase (PC) inhibitor (an inhibitor of furin and other members of PC, including PC1/3, PC4, PAEC4 and PC5/6). The WO group and T+O group without addition of protease inhibitors included 12 extra biopsies for later extractions. The cellular viability of explants was tested by the AlamarBlue assay at baseline, in addition to day 7, 14 and 21(S3 Fig). The media were changed every 2 or 3 days until the last day with fresh medium containing the same reagents as those used at the beginning. The collected supernatants of 9 time points were stored at -20°C for biomarkers analysis.

### Cartilage explants extracts

Extractions were performed by mean of PronaseE/collagenase as previously described [15] in order to quantify the amounts of active ADAMTS-4, ADAMTS-5, MMP-13 and MMP-9 retained in the cartilage matrix. For this purpose, four replicates of cartilage explants from the WO group and T+O were taken out from each day 7, 14 and 21. The enzymatic reactions were stopped using EDTA (in PBS) before applying the extracts for biomarker measurements. The enzyme solutions alone were used as negative control (data not shown).

### Development and characterization of the antibodies against active ADAMTS-5 and MMP-13

Monoclonal antibodies were developed with the same procedure as previously described for the active ADAMTS-4 specific antibody [15]. Full length ADAMTS-5 is an inactive zymogen that is activated by furin-like propeptide convertase cleavage at R^262^↓^263^S [19]. The free N-terminus of active ADAMTS-5 containing SISRARQVEL sequence was chosen as a target for the antibody development and conjugated with maleimide-activated keyhole-limpet hemocyanin (KLH, Thermo Fish, Beijing, China) as immunogen. Pro-MMP-13 is processed by MMP-2 and membrane-type metalloproteinase (MT1-MMP) at E^84^↓Y^85^Tyr to achieve activity [20]. The exposed N-terminus of active MMP-13 with the sequence of YNVFPRTLKW is specific for this enzyme and was therefore synthesized and conjugated with KLH as immunogen. Female, six-week-old Balb/c mice were then injected subcutaneously (s.c.) with 60μg of the immunogens emulsified with Freund’s complete adjuvant in equal volume. One week later, two s.c. injections with 30μg immunogen emulsified with Freund’s incomplete adjuvant were given 2 weeks apart, followed by four s.c. injections every-3-week. Mice bloods were taken since the third immunization. The 4^th^ bleed sera were titrated against the corresponding immunogen and the mouse with the highest titration was chosen for fusion. The standard method was used to produce monoclonal antibodies [21].

The monoclonal antibodies were tested for the specificities against a selection peptide, a elongated peptide (one aa extended at the N-terminus of specific peptide), a cross-reaction peptide, purified pro-enzyme (regarding ADAMTS-5, only active recombinant form available) and a APMA-activated form of MMP-13 (activation at 37°C for 2hr were tested). Moreover, the pro-form and the active form of ADAMTS-5, MMP13 and supernatants of T+O stimulated bovine cartilage explants were subjected to 4–12% gradient SDS-PAGE gel (Life technologies, Denmark) and transferred to nitrocellulose membranes. The membranes were incubated with the monoclonal antibodies. Blots were developed using the enhanced chemiluminescence (ECL) kit (GE healthcare, Denmark).

### Development of the active ADAMTS-5 and MMP-13 ELISAs

Competitive ELISAs were developed for estimation of active ADAMTS-5 and active MMP-13 by applying the developed antibodies targeting active ADAMTS-5 or active MMP-13. Key reagents, such as concentration, buffer, incubation time and temperature were optimized. The final assay protocol was described briefly: 100μl biotinylated peptide was added to a streptavidin pre-coated plate and incubated at 20°C for 30 min. Next, the plate was washed 5 times with standard wash buffer. 20μl standards or samples together with 100μl peroxidase labeled antibody were added to the plate and incubated at 20°C for 2hr (for active ADAMTS-5 assay) or 4 hr (for active MMP-13 assay) with shaking. Afterwards, the wells were washed 5 times and 100 μl/well 3,3’,5,5’-tetramethylbenzidine (TMB) was added and incubated in the dark at 20°C for 15 min. Lastly, 100μl/well stopping solution (0.1% H_2_SO_4_) was added and the colorimetric reaction was measured at 450nm with reference at 650nm.

### The active MMP-9 assay

This novel assay specifically detects a neo-epitope, FQTFEGDLKW, in the N-terminus of active MMP9 (unpublished). The protocol for active MMP9 was described in detail: 100μl biotinylated coater was added to a streptavidin pre-coated plate and incubated at 20°C for 30 min. The plate was washed 5 times in standard wash buffer. 20μl standards or samples together with 100μl peroxidase labeled antibody was added to the plate and incubated at 20°C for 4 hr with shaking. After that, the wells were washed 5 times and 100 μl/well 3,3’,5,5’-tetramethylbenzidine (TMB) was added and incubated in the dark at 20°C for 15 min. Finally, 100μl/well stopping solution (0.1% H_2_SO_4_) was added and the colorimetric reaction was measured at 450nm with reference at 650nm

### The active ADAMTS-4 assay

Active ADAMTS-4 was measured by using a newly developed ELISA assay as previously described [15]. This assay is based on a monoclonal antibody targeting the sequence (FASLSRFVET) at the N-terminus of active ADAMTS-4. In brief the protocol was as follows: The streptavidin pre-coated microtiter plate was coated with 100μL biotinylated coater for 30 min at 20°C with shaking. The plate was then washed 5 times with standard wash solution. 20 μl/well standards or samples were added to the plate, followed by the addition of 100 μl/well peroxidase-labeled antibody and incubated overnight (20±1 h) at 4°C with shaking. Afterwards, the wells were washed 5 times and incubated with 100 μl/well Enhanced 3,3’,5,5’-tetramethylbenzidine (TMB) in the dark at 20°C for 15 min. Finally, 100 μl/well stopping solution was added and the colorimetric reaction was measured at 450 nm with reference at 650 nm.

### The AGNxI and AGNxII assays

The development of AGNxI and AGNxII assays were described in our previous study [16,17]. The competitive AGNxI ELISA assay was developed based on a monoclonal antibody, 1H11, specific for an aggrecanase-derived aggrecan neo-epitope NITEGE^373^.

The sandwich ELISA assay, AGNxII, was previously developed by employing a monoclonal antibody, F-78, against G2 domain as capture antibody and a monoclonal antibody AF-28 recognizing the aggrecan neo-epitope ^342^FFGVG generated by MMP cleavage as detection antibody. The supernatants of all groups were 60x pre-diluted before added to the assay plate.

### The CTX-II assay

The commercial available CTX-II assay detects the C-telopeptide of type II collagen based on a monoclonal antibody specific for a fragment EKGPDP. This assay was performed strictly to follow the manufacturer’s protocol (IDS, UK). The supernatants of all groups were 2000x pre-diluted before added to the assay plate.

The technical performance of each assay is listed in table 1.

**Table 1:** Overview of technical performance of the ELISA assays.

Assay name	Target	Detection Range (ng/ml)	Lower Detection of Limit (ng/ml)	Intra-assay CV(%)	Inter-assay CV(%)
active ADAMTS-4 (He et al., 2013)	N-terminus of active ADAMTS-4	0.1–11.6	0.01	5.4	8.9
active ADAMTS-5	N-terminus of active ADAMTS-5	0.1–8.8	0.08	4.8	8.0
active MMP-13	N-terminus of active MMP-13	0.2–5	0.10	14.7	15.9
active MMP-9 (unpublished)	N-terminus of active MMP-9	0.1–7.1	0.10	9.5	10.3
AGNxI (Sumer et al., 2007;Wang et al., 2009)	Aggrecanase-derived aggrecan neo-epitope	3.4–245.8	1.7	3.8	7.2
AGNxII (Sumer et al., 2007;Wang et al., 2009)	MMP-derived aggrecan neo-epitope	33.3–2110.2	19.5	3.6	8.5
CTX-II (IDS, UK)	MMP-derived type II collagen fragments	1.2–300 (pg/ml)			

The technical performance of assayed used in this study was summarized and listed in this table. The lower limit of detection (LLOD) was computed as 3 Standard Deviation (SD) of the mean value of 21 zero standards. Intra-assay CV% (Intra-assay coefficient of variation, within a plate) and Inter-CV% (inter-assay coefficient of variation, between different plates) were calculated as the mean value of the variation of 8 samples analyzed 10 times in duplicate.

### Statistics

Biomarker levels were shown as mean ± 95% confidence interval (CI) (n = 8 in each group). Differences in biomarker levels between the WO group and T+O group on 9 time points were assessed using the non-parametric Mann-Whitney *U*-test, since the assumption of normality has been violated in *t*-test (small sample sizes). 2-way ANOVA was used to test the effects of inhibitors followed by post-hoc Bonferroni pair wise comparisons to correct for multiple comparisons from multiple inhibitors treatments. Differences were considered statistically significant if P<0.05 and significant levels were displayed as: * = P<0.05; ** = P<0.01, *** = P<0.001 and **** = P<0.0001Statistical analysis was performed using GraphPad Prism 6.

## Results

### Characterization of the novel active ADAMTS-5 and MMP-13 antibodies

**Fig 1:**
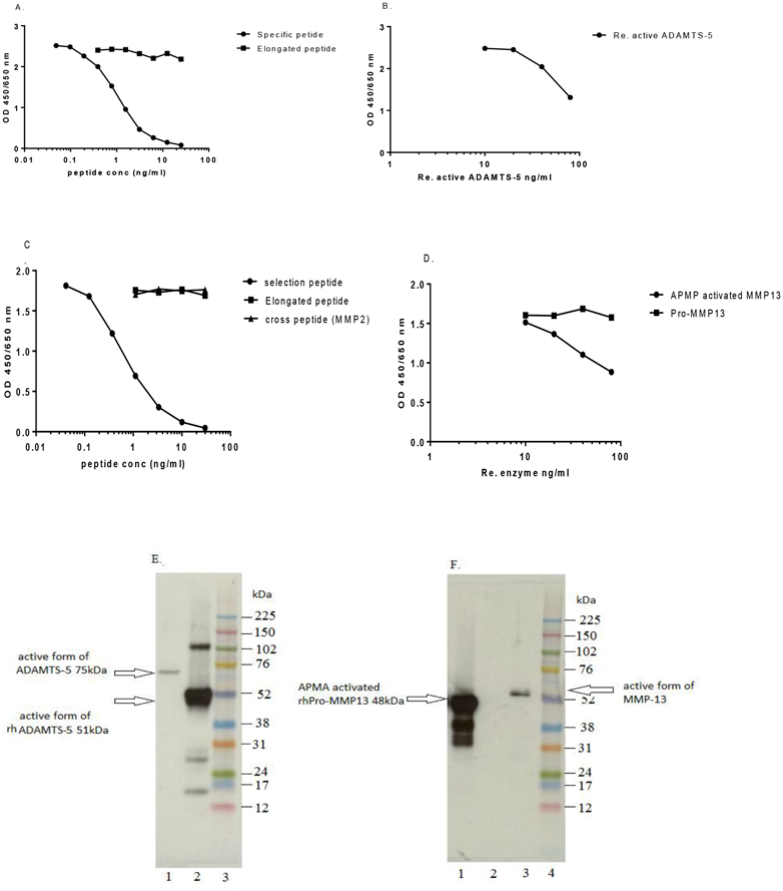
Characterization of active ADAMTS-4 antibody, NB421-13F7, and active MMP-13 antibody, NB469-8C11. (A) The binding of NB421-13F7 to biotinylated peptide was displaced by a specific peptide (SISRARQVEL), but not a elongated peptide (RSISRARQVEL). (B) Displacement of signal with the recombinant human ADAMTS-5 (starting from 80ng/ml to 10ng/ml in2-fold dilution steps) (C) The binding of NB469-8C11 to biotinylated peptide was displaced by a specific peptide (YNVFPRTLKW), but neither a elongated peptide (EYNVFPRTLKW) nor a cross peptide (YNFFPRKPKW, MMP2). (D) Displacement of signal by APMA-activated MMP-13form, but not Pro-MMP13 (starting from 80ng/ml to 10 ng/ml in 2-fold dilution steps). (E) Western blot of active ADAMTS-5 in the supernatant of T+Oinduced bovine cartilage explants (Lane1) and recombinant active ADAMTS-5(Ser^262^-Pro^622^) (Lane2). (F)Western Blot of active MMP-13 in APMA-activated recombinant of MMP-13 (Lane1) and the supernatant of T+O induced bovine cartilage explants, but not, recombinant Pro-MMP13 (Lane 2).

The signal of the antibody NB421-13F7 (isotype: IgG2a, κ) was displaced by increasing concentrations of specific peptide (SISRARQVEL) and recombinant human ADAMTS-5 (Ser^262^-Pro^622^), but not by the N-terminal elongated peptide (RSISRARQVEL) (Fig 1A–1B). A single band of 75kDa was seen in the supernatant of T+O stimulated bovine cartilage explants by western blot analysis. A major band of 51 kDa was observed in the recombinant active ADAMTS-5 protein (Ser^262^-Pro^622^) (Fig 1E).

A similar displacement experiment showed that the signal of NB469-8C11 (isotype: IgG2a, κ) was inhibited by the specific peptide (YNVFPRTLKW) and APMA-activated form of MMP-13. However, the elongated peptide (EYNVFPRTLKW), the cross-reaction peptide of MMP2 (YNFFPRKPKW) and recombinant Pro-MMP-13 were unable to block the signal (Fig 1C–1D). A major 48kDa band and two smaller bands were observed in APMA-activated Pro-MMP-13 products, which could be the full length of active MMP-13, as well as further degradation on C-terminus of active MMP-13. No bands were seen for Pro-MMP-13. A single band around 51kDa was found in the supernatant of T+O stimulated bovine cartilage explants (Fig 1F). These data indicate that NB421-13F7 and NB469-8C11 are specific for active form of ADAMTS-5 and MMP-13, respectively.

### Profiling of active aggrecanases and their specific aggrecan degradation fragments in TNF-α/OSM stimulated explants

According to the AlamarBlue data (S1 Fig), the cell viability of the harvested explants stabilized after 7 days in serum-free medium, but had a tendency to decrease gradually over time from 14823 (OD value) to 11461 at day 21,although these changes were not statistically significant.

**Fig 2:**
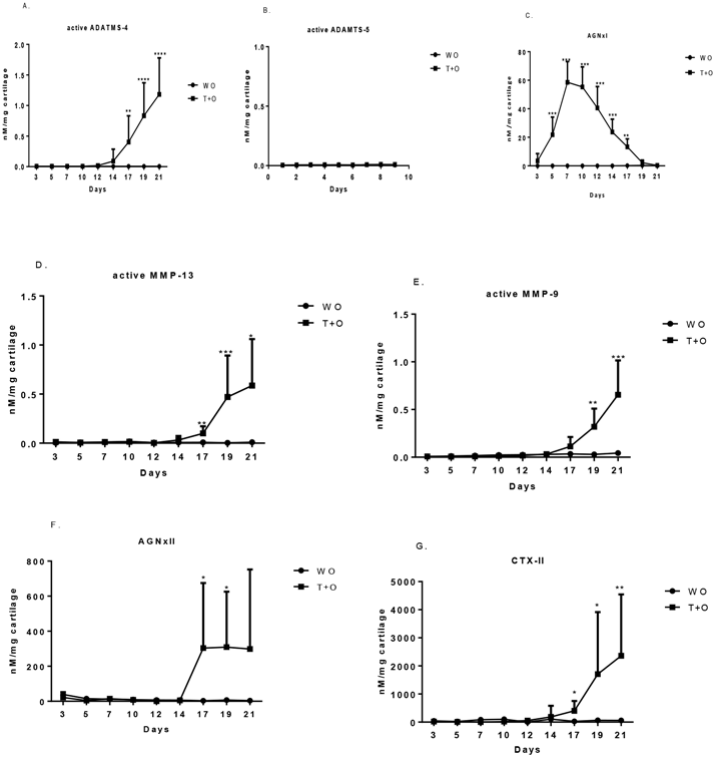
Profiling of active aggrecanases, MMPs and their specific aggrecan degradation fragments into bovine explants upon T+O stimulation. The releases of (A)active ADAMTS-4.: (D) active MMP-13, (E) active MMP-9, (F)AGNxII and (G)CTX-II started since day 14 and increased over time, while (B) active ADAMTS-5 was unable to be detected during the 21 days. (C) Aggrecanase-mediated aggrecan fragment’s levels peaked at day 7, afterwards, declined to background levels at day 21. No any release of any biomarkers was seen in the WO group. All values were shown as mean ±95% confidence intervals (n = 8 in each group). Error bars indicated the upper limit of 95% confidence intervals.

**Fig 3:**
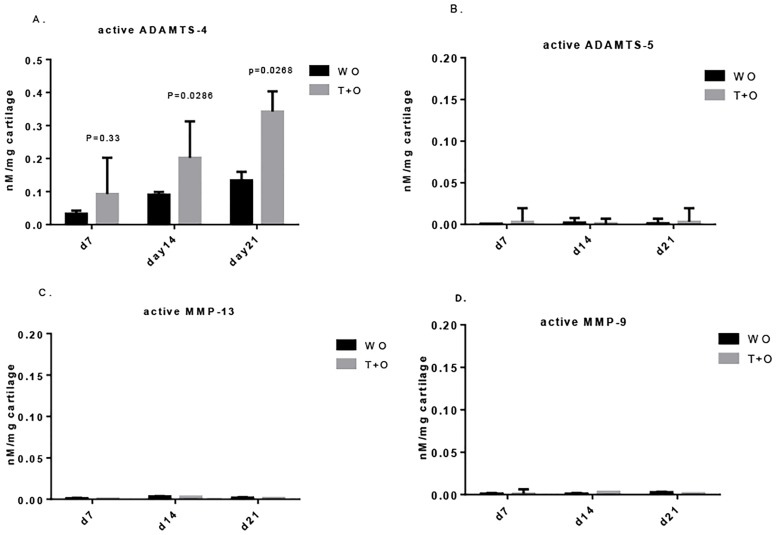
The cartilage extracts by enzyme degradation method from WO group and T+O group were tested for 4 active proteases. Cartilage explants from WO group and T+O group at day 7, 14 and 21 were extracted by using mean of enzymes as previously described [15]. (A) active ADAMTS-4 was accumulated in the WO group. However, stimulation of T+O significantly induced the elevated level of active ADAMTS-4 retained in the matrix. None of (B) active ADAMTS-5, (C) active MMP-13, (D) active MMP-9 was detected in untreated group or treated group. All values were shown as mean±95% confidence intervals (n = 4 in each group). Error bars indicated the upper limit of 95% confidence intervals.

In WO cultures, no active ADAMTS-5, MMP13 or MMP9 were detected in the media (Fig 2B, 2D and 2E) or accumulated in the tissue (Fig 3B–3D). In addition, no active ADAMTS-4 was released into the medium (Fig 2A), but active ADAMTS-4 was accumulated in the tissue over time (Fig 3A). In the T+O explants, active ADAMTS-4, MMP-13 and MMP9 were all released in the media, beginning from day 14 and then increasingly throughout the end of the experiment at day 21 (Fig 2A, 2D and 2E). In the WO group, active ADAMTS-4 alone was accumulated in the tissue (Fig 3A). 89.6% of the total active ADAMTS-4 was released based on the calculation of area under curve (AUC) (Figs 2A and 3A). ADAMTS-5 acted differently from the other active enzymes as no tissue accumulation or release into medium was detected no matter with or without catabolic stimulation (Figs 2B and 3B). When aggrecan degradation was evaluated by mean of the AGNxI assay, the aggrecanase-derived neo-epitope NITEGE^373^, was found to peak at day 10, and then declined afterwards (Fig 2C).

### Profiling of active MMPs and their specific aggrecan and collagen degradation fragments in TNF-α/OSM stimulated explants

Both active MMP-13 and -9 were released from day 17 and peaked at day 21(Fig 2D and 2E). The release of MMP-generated aggrecan fragments (AGNxII) and type II collagen fragments (CTX-II) increased over time and peaked in the late stage (day 17–21) (Fig 2F and 2G). Large variations have been seen in AGNxII and CTX-II measurements. It seems likely that the variation of the results originates from the biological differences between biopsies.

### The effect of broad-spectrum inhibitors on the profiling of active aggrecanases and their specific aggrecan degradation fragments

**Fig 4:**
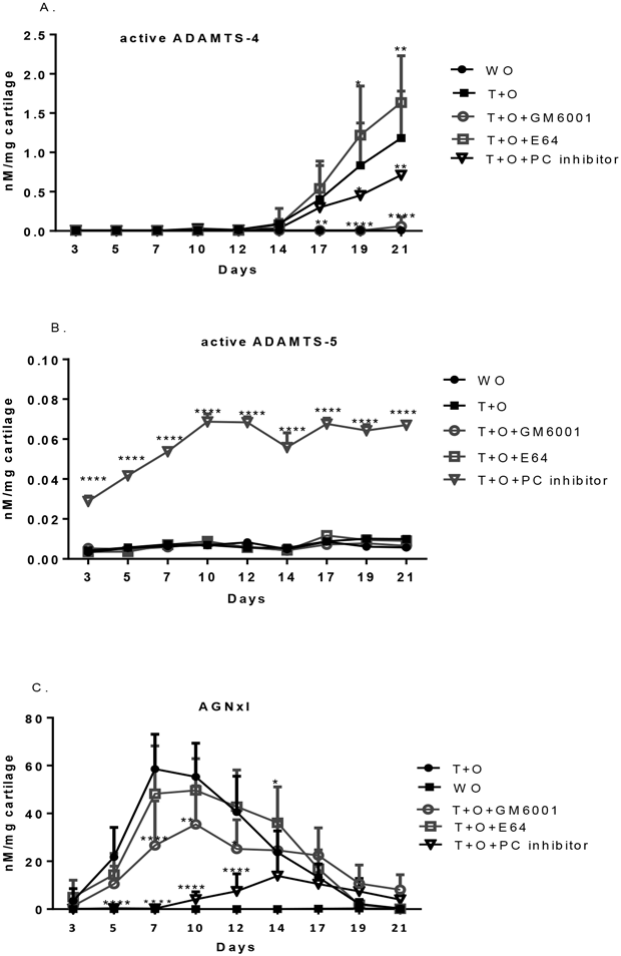
The effect of broad-spectrum inhibitors on the profiling of active aggrecanases and their specific aggrecan degradation fragments. (A) active ADAMTS-4. (B) active ADAMTS-5. (C) AGNxI. All values were shown as mean±95% confidence intervals (n = 8 in each group). Error bars indicated the upper limit of 95% confidence intervals.

Co-incubation of GM6001 with T+O media almost completely inhibited the release of active ADAMTS-4. Supplement of E64 to the T+O media let to an increased level of active ADAMTS-4 compared to the group treated with T+O alone. Furin-like PC inhibitor blocking the removal of pro-domain from ADAMTS- 4 [22] resulted in a delay and decreased release of active ADAMTS-4 (Fig 4A).

Completely different results were seen for active ADAMTS-5. The PC inhibitor was the only one of the tested conditions that remarkably altered the release of active ADAMTS-5. The PC inhibitor induced rather than suppressed a high level of active ADAMTS-5 (Fig 4B).

GM6001 partially blocked the release of AGNxI (ADAMTS-mediated aggrecan degradation), while the PC inhibitor induced a significant decrease and delay in the release of AGNxI. There was no significant impact of E64 observed on the profile of AGNxI (Fig 4C).

### The effect of broad-spectrum inhibitors on the profiling of active MMPs and their specific aggrecan and collagen degradation fragments

**Fig 5:**
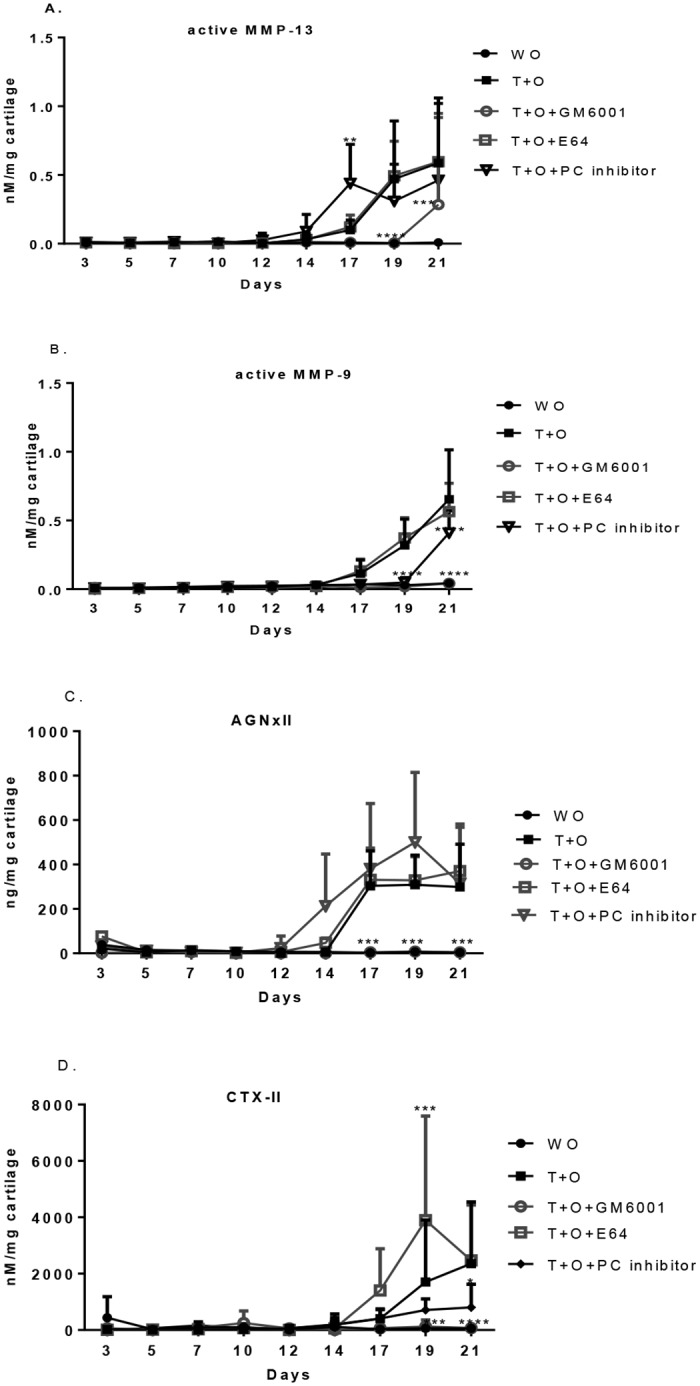
The effect of broad-spectrum inhibitors on the profiling of active MMPs and their specific aggrecan and collagen degradation fragments. (A) active MMP-13. (B) active MMP-9. (C) AGNxI. (D) CTX-II. All values were shown as mean±95% confidence intervals (n = 8 in each group). Error bars indicated the upper limit of 95% confidence intervals.

As expected, GM6001 almost blocked the release of active MMP-13 and -9, since the activation of both latent Pro-MMP-13, -9 is matrix metalloproteinase-dependent. E64 did not significantly change the release pattern of active MMP-13 and -9. The PC inhibitor induced a significant earlier release of active MMP-13, while it significantly suppressed the release of active MMP-9 (Fig 5A–5B).

MMP-dependent aggrecan degradation measured by AGNxII was completely inhibited by GM6001. In contrast, the PC inhibitor induced significant release of AGNxII as early as day14. E64 had no effect on AGNxII (Fig 5C).

Type II collagen degradation assessed by CTX-II remained at background levels at all time points when GM6001 was added. Its release was significantly inhibited by PC inhibitor over time. Interestingly, E64 caused early and more release of CTX-II (Fig 5D).

### Area under the biomarker level vs. time curve (AUC)

The cumulative effects of the various inhibitors on biomarkers levels during the 21 days were calculated by AUC and displayed as the percent change in AUC compared to the T+O treatment (Table 2). A ±15% change caused by inhibitor in AUC was defined as no alteration, considering the acceptable inter-assay variation of biomarkers. The broad MMP inhibitor, GM6001, almost completely inhibited the activation of Pro-ADAMTS-4, Pro-MMPs and cartilage degradation. Blockade of cysteine proteases by E64 increased the level of active ADAMTS-4 and the release of type II collagen fragments. The PC inhibitor dramatically lowered the level of active ADAMTS-4 and aggrecanase-mediated degradation, but induced a huge release of active ADAMTS-5 instead to compensate the loss of ADAMTS-4. The PC inhibitor also increased the conversion of latent MMP-13 to active form and the release of AGNxII.

**Table 2:** Area under the biomarker level vs. time curve (AUC).

		WO (n = 8)	T+O (n = 8)	T+O+GM6001 (n = 8)	T+O+E64(n = 8)	T+O+PCi (n = 8)
**ADAMTS-4**	**Mean**	0.11	3.60	0.21	5.17	2.70
	**95% CI (lower limit; upper limit)**	(0.11; 0.11)	(1.72; 5.48)	(0.04; 0.37)	(2.50; 7.84)	(0.50; 4.90)
	**% change in AUC**	-	0	**↓** 94.2%	**↑** 43.6	**↓** 25
**DAMTS-5**	**Mean**	0.12	0.13	0.11	0.13	1.06
	**95% CI (lower limit; upper limit)**	(0.11; 0.13)	(0.11; 0.14)	(0.10; 0.12)	(0.11; 0.15)	(1.00; 1.11)
	**% change in AUC**	-	0	**.** 13.2	**.** 0.8	**↑** 721.7
**AGNxI**	**Mean**	1.06	509.9	374.8	500.5	106.4
	**95% CI (lower limit; upper limit)**	(0.35; 1.77)	(400.7; 619.1)	(280.8;468.8)	(355.6; 645.4)	(61.08; 151.8)
	**% change in AUC**	-	0	**↓** 26.4	**.** 1.8	**↓ 7**9.1
**MMP-13**	**Mean**	0.15	1.95	0.42	2.00	2.53
	**95% CI (lower limit; upper limit)**	(0.13; 0.17)	(0.74; 3.16)	(-0.22; 1.05)	(1.18; 2.85)	(1.29; 3.8)
	**% change in AUC**	-	0	**↓** 78.6	**.** 2.3	**↑** 29.8
**MMP-9**	**Mean**	0.45	1.76	0.26	1.81	0.76
	**95% CI (lower limit; upper limit)**	(0.35; 0.54)	(1.01;2.51)	(0.21;0.31)	(1.16;2.46)	(0.54;0.98)
	**% change in AUC**	-	0	**↓** 85.1	**.** 2.7	**↓** 56.8
**AGNxII**	**Mean**	526.1	1432	384.9	1724	2919
	**95% CI (lower limit; upper limit)**	(-286.1; 1338)	(-243.4; 3108)	(-421.7; 1191)	(288.7; 3159)	(1210; 4628)
	**% change in AUC**	-	0	**↓** 73.1	**↑**20.4	**↑** 103.8
**CTX-II**	**Mean**	1480	6844	2730	13165	4606
	**95% CI (lower limit; upper limit)**	(-37.94; 2997)	(830.9; 12857)	(-5,054,954)	(4917; 21414)	(2117; 7095)
	**% change in AUC**	-	0	**↓** 60.1	**↑** 92.4	**↓** 32.7

AUC means area under the biomarker level vs. time curve, representing the cumulative effect of various inhibitors on biomarker levels over total 21 days. AUC values were shown as mean± 95% confidence intervals (lower limit; upper limit). The percent changes in AUC value after the addition of inhibitors were compared with only TNF-α plus OSM. A ±15% change caused by inhibitor in AUC value was defined as no alteration and indicated by “∙”, considering the acceptable variability in the measurements of biomarkers.

## Discussion

**Fig 6:**
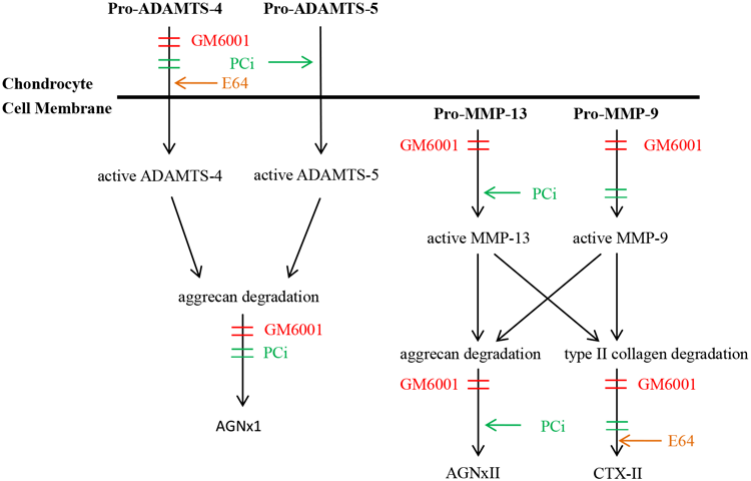
The schematic diagram of the effects of inhibitors on protease activation and cartilage degradation. The broad MMP inhibitor, GM6001 (red color), almost completely inhibited the activation of Pro-ADAMTS-4, Pro-MMPs and cartilage breakdown, since the efficient gaining of activities of both pro-enzymes is MMP-dependent process. Addition with Furin-like convertase inhibitor, PCi (green color), decreased the release of active ADAMTS-4 and AGNxI induced by T+O, but induced huge release of active ADAMTS-5, suggesting active ADAMTS-4 is the predominant aggrecanase for the generation of AGNxI in T+O treated-bovine cartilage explants. PCi promoted the activation of MMP-13 and AGNxII, but not MMP-9. Blockade of cysteine proteases by E64 increased the levels of active ADAMTS-4 and the release of CTX-II, whereas had little effect on the activation of MMP-13 and MMP-9, demonstrating that other compensatory collagenases possibly were triggered following by the inhibition of cysteine protease activity. In summary, the activation of pro-enzyme is a key point in cartilage degradation of T+O treated bovine cartilage, which is regulated in an integrated cascade process.

Neo-epitope antibodies recognize the products of a proteolytic event and therefore have been widely used as markers of enzyme activities. For example, the neo-epitope antibodies specific for ^374^ARGS and ^342^FFGVG have been successfully employed to clarify the distinct roles of aggrecanases and MMPs in human joint aggrecanolysis [23,24]. However, neo-epitope antibodies do not distinguish the activities between ADAMTS-4 and ADAMTS-5, both of which can generate ^374^ARGS fragment, or several MMPs when they cleave type II collagen at the same cleavage site. The neo-epitope antibody approach has also been used to detect the N-terminal sequence of furin-like activated ADAMTS-4 or ADAMTS-5 in other studies [25,26]. To our knowledge, this is the first study to detect aggrecanases and MMPs directly by using neo-epitope antibodies in catabolic factors stimulated bovine cartilage explants and to gain an overview of the profiling of these key enzymes. We found that ADAMTS-4 was the major protease for the IGD cleavage of aggrecan in the presented study. Furthermore, the compensatory mechanism might be triggered following the blockage or down-regulation the activity of one enzyme (Fig 6).

Active ADAMTS-4 was accumulated in the tissue for about 12 days before any release was detectable and the level of active ADAMTS-4 released in the medium plus that retained in tissue increased continuously during the 21 days (Figs 2A and 3A). The aggrecanase-derived neo-epitope NITEGE^373^ measured by AGNxI assay peaked at day 10, but declined afterwards (Fig 2C). This pattern, where the release of active ADAMTS-4 is initiated after the majority of aggrecan has been degraded, has been identified in our previous study as well [15]. Two other groups had similar findings when they applied ADAMTS-4 transfected human chondrosarcoma cells [27] or IL-1 treated bovine cartilage tissues [28]. The probable explanation for the delay as well as for the absence of active ADAMTS-5 in medium or extracts (Figs 2B and 3B) is that the liberation of active ADAMTS-4 remained associated in the matrix.

ADAMTS-4 and ADAMTS-5 are synthesized as zymogens and the N-terminal processing mediated by furin-like proprotein convertase (PC) is essential for ADAMTS-4 and -5 gaining activity. Intracellular [22], cell surface [29] and extracellular [30] activation have been reported for ADAMTS-4, whereas ADAMTS5 can be extracellularly activated [19,30]. The release of active ADAMTS-4 after day 12 was totally blocked by the metalloproteinase inhibitor, GM6001. It’s probably because an efficient activation of pro-ADAMTS-4 involves MT4-MMP activity[27,28]. The partial effect of the PC inhibitor might result from diffusion constraints of this molecule in the cartilage (Fig 4A).

The response of ADAMTS-5 expression to inflammatory factors has been investigated in many studies, but contradictory results have been reported [31–34]. As summarized by Fosang *et al*., variation in culture conditions, primary cell or transformed cell, health status of explants, the species can lead to huge differences in the outcome [35]. The markedly enhanced release of active ADAMTS-5 by the PC inhibitor (Fig 4B) is either the result of compensating the block of ADAMTS-4 or from its matrix-bound form. The later is unlikely to happen, since active ADAMTS-5 was undetected in extracts of T+O alone explants (Fig 3B). Further investigation is required for validation of this interesting finding.

Taken together, GM6001 and PC inhibitor blocked the release of active ADAMTS-4 and concurrently blocked the release of AGNxI (NITEGE^373^), whereas the release of active ADAMTS-5 increased in the presence of PC inhibitor as mentioned above. The data represent strong evidence on the central role of ADAMTS-4 in aggrecanolysis in this experimental system.

Milner *et al*. demonstrated that collagen release was rarely seen before day 7 in IL-1α/OSM treated-bovine cartilage explants and the activation of pro-collagenase was a rate-limiting step in bovine cartilage breakdown[14]. Our data suggest that both active MMP-13 and MMP-9 were detectable in the late stage of TNF-α/OSM stimulation only (Fig 2D and 2E). We found that their specific cleavage aggrecan and collagen fragments, AGNxII and CTX-II respectively (Fig 2F and 2G), were released at day 17 and increased correspondingly with the increased level of active MMP-13 and MMP-9. The degradation events involving the MMPs occurred after the ADAMTS-mediated cartilage degradation.

Co-incubation with the MMP broad-spectrum inhibitor, GM6001, almost fully inhibited the level of active MMP-13, MMP-9 and partly blocked the release of AGNxII and CTX-II due to blocking of the activation pathway (Fig 5A–5B). The PC inhibitor was in favor of the activation of MMP-13 and MMP-derived aggrecan degradation (AGNxII), but weakened the activation of both MMP-9 and CTX-II. The distinct response of active MMP-13 and MMP-9 to inhibitors remains unclear, although their cascades leading to activation are similar to each other. Many studies have revealed that several converging pathways of pro-MMP-13 and -9 activation are likely to co-exist [36–38]. The activation of MT1-MMP by furin and the generation of plasmin from plasminogen by trypsin-like protease, such as urokinase plasmingoen activator (uPA) can initiate the activation of Pro-MMP-13 and -9.

The blockage of cysteine protease activity such as cathepsin K by E64 leaded to an increased release of active ADAMTS-4 and CTX-II (Figs 4A and 5D). In line with the results, Sondergaard *et al*. showed that inhibition of cysteine protease activity by E64 in TNF-α/OSM stimulated bovine cartilage explants resulted in an increase of CTX-II level, and more, this up-regulation was observed *in vivo* CK null mice as well [39]. We further showed, in this study, that active MMP-13 and MMP-9 were not likely the cause of overproduction of CTX-II fragments, since their levels remained the same, but other compensatory collagenases possibly were triggered following by the inhibition of cysteine protease activity.

## Conclusions

We found that ADAMTS-4 appeared to be the major protease for the generation of ^374^ARGS aggrecan fragment in the TNF-α/OSM stimulated bovine cartilage explants. This study reveals the need to determine the roles of ADAMTS-4 and ADAMTS-5 in human articular degradation during OA and hence identify the attractive target for slowing down human cartilage breakdown. In addition, this study aids in the design of inhibitors of key enzymes for therapeutic intervention in OA.

## Supporting Information

S1 Fig The cell viability of explants in serum-free medium was measured by Alamar blue. the cell viability of the harvested explants stabilized after 7 days in serum-free medium, but had a tendency to decrease gradually over time from 14823 (OD value) to 11461 at day21, although these changes were not statistically significant.
(TIF)

S2 Fig The Safranin O/Fast green staining of bovine cartilage cultured in serum-free medium. There was no significant loss of the proteoglycan of three zones (upper, middle and deep zones) after 21 days culture in serum-free medium
(TIF)

S3 Fig The cell viability of explants from different groups was measured by Alamar blue assay on baseline, 7, 14 and 21 days. All values were shown as mean±95% confidence intervals (n = 8 in each group). Error bars indicated the upper limit of 95% confidence intervals. The cell viability of the harvested explants stabilized after 7 days culture in serum-free medium, and then decreased gradually over time, although these changes were not statistically significant. The cellular viability was not affected by stimulators, inhibitors or vehicle after 7 days until it dropped significantly at day 14 since there was cartilage degradation induced by stimulators. The addition of inhibitors improved the cell viability to some extent compared to T+O alone group. On day 21, the cell viabilities of T+O with or without inhibitors groups were significantly lower than the WO group, but no significant difference was detected among the groups. The data indicated that the distinct responses of biomarkers in each group resulted from the changes in the metabolism instead of the viability of chondrocytes caused by the inhibitors.
(TIF)

## References

[1] Bitton R. The economic burden of osteoarthritis. Am J Manag Care. 2009; 15: S230–235. 19817509

[2] Garnero P, Ayral X, Rousseau JC, Christgau S, Sandell LJ, Douqados M, et al Uncoupling of type II collagen synthesis and degradation predicts progression of joint damage in patients with knee osteoarthritis. Arthritis Rheum. 2002; 46: 2613–2624. 12384919 10.1002/art.10576

[3] Tortorella MD, Pratta M, Liu RQ, Austin J, Ross OH, Abbaszade I, et al Sites of aggrecan cleavage by recombinant human aggrecanase-1 (ADAMTS-4). J Biol Chem. 2000; 275: 18566–18573. 10751421 10.1074/jbc.M909383199

[4] Tortorella MD, Liu RQ, Burn T, Newton RC, Arner E. Characterization of human aggrecanase 2 (ADAM-TS5): substrate specificity studies and comparison with aggrecanase 1 (ADAM-TS4). Matrix Biol. 2002; 21: 499–5115. 12392761 10.1016/s0945-053x(02)00069-0

[5] Mercuri FA, Maciewicz RA, Tart J, Last K, Fosang AJ. Mutations in the interglobular domain of aggrecan alter matrix metalloproteinase and aggrecanase cleavage patterns. Evidence that matrix metalloproteinase cleavage interferes with aggrecanase activity. J Biol Chem. 2000; 275: 33038–33045. 11032846 10.1074/jbc.275.42.33038

[6] Wang M, Sampson ER, Jin H, Li J, Ke QH, Im HJ, et al MMP13 is a critical target gene during the progression of osteoarthritis. Arthritis Res Ther. 2013; 15: R5 10.1186/ar4133 23298463 PMC3672752

[7] Neuhold LA, Killar L, Zhao W, Sung ML, Warner L, Kulik J, et al Postnatal expression in hyaline cartilage of constitutively active human collagenase-3 (MMP-13) induces osteoarthritis in mice. J Clin Invest. 2001; 107: 35–44. 11134178 10.1172/JCI10564 PMC198546

[8] Wang X, Manner PA, Horner A, Shum L, Tuan RS, Nuckolls GH. Regulation of MMP-13 expression by RUNX2 and FGF2 in osteoarthritic cartilage. Osteoarthritis Cartilage. 2004; 12: 963–973. 15564063 10.1016/j.joca.2004.08.008

[9] Galasso O, Familiari F, De Gori M, Gasparini G. Recent findings on the role of gelatinases (matrix metalloproteinase-2 and -9) in osteoarthritis. Adv Orthop 2012 2012: 834208 10.1155/2012/834208 22900195 PMC3412089

[10] Karsdal MA, Sumer EU, Wulf H, Madsen SH, Christiansen C, Fosang AJ, et al Induction of increased cAMP levels in articular chondrocytes blocks matrix metalloproteinase-mediated cartilage degradation, but not aggrecanase-mediated cartilage degradation. Arthritis Rheum. 2007; 56: 1549–1558. 17469134 10.1002/art.22599

[11] Hui W, Rowan AD, Richards CD, Cawston TE. Oncostatin M in combination with tumor necrosis factor alpha induces cartilage damage and matrix metalloproteinase expression in vitro and in vivo. Arthritis Rheum. 2003; 48: 3404–3418. 14673992 10.1002/art.11333

[12] Bay-Jensen AC, Hoegh-Madsen S, Dam E, Henriksen K, Sondergaard BC, Pastoureau P, et al Which elements are involved in reversible and irreversible cartilage degradation in osteoarthritis? Rheumatol Int. 2010; 30: 435–442. 10.1007/s00296-009-1183-1 19816688

[13] Karsdal MA, Madsen SH, Christiansen C, Henriksen K, Fosang AJ, Sondergaard BC. Cartilage degradation is fully reversible in the presence of aggrecanase but not matrix metalloproteinase activity. Arthritis Res Ther. 2008; 10: R63 10.1186/ar2434 18513402 PMC2483454

[14] Milner JM, Elliott SF, Cawston TE. Activation of procollagenases is a key control point in cartilage collagen degradation: interaction of serine and metalloproteinase pathways. Arthritis Rheum. 2001 44: 2084–2096. 11592371 10.1002/1529-0131(200109)44:9<2084::AID-ART359>3.0.CO;2-R

[15] He Y, Zheng Q, Simonsen O, Petersen KK, Christiansen TG, Karsdal MA, et al The development and characterization of a competitive ELISA for measuring active ADAMTS-4 in a bovine cartilage ex vivo model. Matrix Biol. 2013; 32: 143–151. 10.1016/j.matbio.2012.12.001 23295731

[16] Wang B, Chen P, Jensen AC, Karsdal MA, Madsen SH, Sondergaard BC, et al Suppression of MMP activity in bovine cartilage explants cultures has little if any effect on the release of aggrecanase-derived aggrecan fragments. BMC Res Notes. 2009; 2: 259 10.1186/1756-0500-2-259 20021645 PMC2803187

[17] Sumer EU, Sondergaard BC, Rousseau JC, Delmas PD, Fosang AJ, Karsdal MA, et al MMP and non-MMP-mediated release of aggrecan and its fragments from articular cartilage: a comparative study of three different aggrecan and glycosaminoglycan assays. Osteoarthritis Cartilage. 2007; 15: 212–221. 16997584 10.1016/j.joca.2006.07.009

[18] Chen-An P, Andreassen KV, Henriksen K, Karsdal MA, Bay-Jensen AC. Investigation of chondrocyte hypertrophy and cartilage calcification in a full-depth articular cartilage explants model. Rheumatol Int. 2013; 33: 401–411. 10.1007/s00296-012-2368-6 22453523

[19] Longpre JM, McCulloch DR, Koo BH, Alexander JP, Apte SS, Leduc R. Characterization of proADAMTS5 processing by proprotein convertases. Int J Biochem Cell Biol. 2009 41: 1116–1126. 10.1016/j.biocel.2008.10.008 18992360

[20] Knauper V, Will H, Lopez-Otin C, Smith B, Atkinson SJ, Stanton H, et al (1996) Cellular mechanisms for human procollagenase-3 (MMP-13) activation. Evidence that MT1-MMP (MMP-14) and gelatinase a (MMP-2) are able to generate active enzyme. J Biol Chem. 1996; 271: 17124–17131. 8663255 10.1074/jbc.271.29.17124

[21] Monoclonal Antibody Production. Washington DC: National Academy of Sciences;1999.

[22] Wang P, Tortorella M, England K, Malfait AM, Thomas G, Arner EC, et al Proprotein convertase furin interacts with and cleaves pro-ADAMTS4 (Aggrecanase-1) in the trans-Golgi network. J Biol Chem. 2004; 279: 15434–15440. 14744861 10.1074/jbc.M312797200

[23] Sandy JD.A contentious issue finds some clarity: on the independent and complementary roles of aggrecanase activity and MMP activity in human joint aggrecanolysis. Osteoarthritis Cartilage. 2006; 14: 95–100. 16257242 10.1016/j.joca.2005.09.004

[24] Fosang AJ, Last K, Stanton H, Golub SB, Little CB, Brown L, et al Neoepitope antibodies against MMP-cleaved and aggrecanase-cleaved aggrecan. Methods Mol Biol. 2010 622: 312–347. 10.1007/978-1-60327-299-5_19 20135291

[25] Mort JS, Flannery CR, Makkerh J, Krupa JC, Lee ER. Use of anti-neoepitope antibodies for the analysis of degradative events in cartilage and the molecular basis for neoepitope specificity. Biochem Soc Symp. 2003; 70: 107–114. 14587286 10.1042/bss0700107

[26] Powell AJ, Little CB, Hughes CE. Low molecular weight isoforms of the aggrecanases are responsible for the cytokine-induced proteolysis of aggrecan in a porcine chondrocyte culture system. Arthritis Rheum. 2007; 56: 3010–3019. 17763444 10.1002/art.22818

[27] Gao G, Plaas A, Thompson VP, Jin S, Zuo F, Sandy JD. ADAMTS4 (aggrecanase-1) activation on the cell surface involves C-terminal cleavage by glycosylphosphatidyl inositol-anchored membrane type 4-matrix metalloproteinase and binding of the activated proteinase to chondroitin sulfate and heparan sulfate on syndecan-1. J Biol Chem. 2004; 279: 10042–10051. 14701864 10.1074/jbc.M312100200

[28] Patwari P, Gao G, Lee JH, Grodzinsky AJ, Sandy JD. Analysis of ADAMTS4 and MT4-MMP indicates that both are involved in aggrecanolysis in interleukin-1-treated bovine cartilage. Osteoarthritis Cartilage. 2005; 13: 269–277. 15780640 10.1016/j.joca.2004.10.023 PMC2771540

[29] Mayer G, Hamelin J, Asselin MC, Pasquato A, Marcinkiewicz E, Tang M, et al The regulated cell surface zymogen activation of the proprotein convertase PC5A directs the processing of its secretory substrates. J Biol Chem. 2008; 283: 2373–2384. 18039650 10.1074/jbc.M708763200

[30] Malfait AM, Arner EC, Song RH, Alston JT, Markosyan S, Staten N, et al Proprotein convertase activation of aggrecanases in cartilage in situ. Arch Biochem Biophys. 2008; 478: 43–51. 10.1016/j.abb.2008.07.012 18671934

[31] Bondeson J, Lauder S, Wainwright S, Amos N, Evans A, Hughes C, et al Adenoviral gene transfer of the endogenous inhibitor IkappaBalpha into human osteoarthritis synovial fibroblasts demonstrates that several matrix metalloproteinases and aggrecanases are nuclear factor-kappaB-dependent. J Rheumatol. 2007; 34: 523–533. 17295438

[32] Vankemmelbeke MN, Holen I, Wilson AG, Ilic MZ, Handley CJ, Kelner GS, et al Expression and activity of ADAMTS-5 in synovium. Eur J Biochem. 2001; 268: 1259–1268. 11231277 10.1046/j.1432-1327.2001.01990.x

[33] Koshy PJ, Lundy CJ, Rowan AD, Porter S, Edwards DR, Hogan A, et al The modulation of matrix metalloproteinase and ADAM gene expression in human chondrocytes by interleukin-1 and oncostatin M: a time-course study using real-time quantitative reverse transcription-polymerase chain reaction. Arthritis Rheum. 2002; 46: 961–967. 11953973 10.1002/art.10212

[34] Chan PS, Caron JP, Orth MW. Short-term gene expression changes in cartilage explants stimulated with interleukin beta plus glucosamine and chondroitin sulfate. J Rheumatol. 2006; 33: 1329–1340. 16821268

[35] Fosang AJ, Rogerson FM, East CJ, Stanton H. ADAMTS-5: the story so far. Eur Cell Mater. 2008; 15: 11–26. 18247274 10.22203/ecm.v015a02

[36] Brinckerhoff CE, Matrisian LM. Matrix metalloproteinases: a tail of a frog that became a prince. Nat Rev Mol Cell Biol. 2002; 3: 207–214. 11994741 10.1038/nrm763

[37] Toth M, Chvyrkova I, Bernardo MM, Hernandez-Barrantes S, Fridman R. Pro-MMP-9 activation by the MT1-MMP/MMP-2 axis and MMP-3: role of TIMP-2 and plasma membranes. Biochem Biophys Res Commun. 2003; 308: 386–395. 12901881 10.1016/s0006-291x(03)01405-0

[38] Ramos-DeSimone N, Hahn-Dantona E, Sipley J, Nagase H, French DL, Quiqley JP. Activation of matrix metalloproteinase-9 (MMP-9) via a converging plasmin/stromelysin-1 cascade enhances tumor cell invasion. J Biol Chem. 1999; 274: 13066–13076. 10224058 10.1074/jbc.274.19.13066

[39] Sondergaard BC, Henriksen K, Wulf H, Oestergaard S, Schurigt U, Bräuer R, et al Relative contribution of matrix metalloprotease and cysteine protease activities to cytokine-stimulated articular cartilage degradation. Osteoarthritis Cartilage. 2006; 14: 738–748. 16563811 10.1016/j.joca.2006.01.016

