# Supplementary figures and images for: The Effect of Protease Inhibitors on the Induction of Osteoarthritis-Related Biomarkers in Bovine Full-Depth Cartilage Explants
Source: PLoS One. 2015 Apr 24;10(4):e0122700. doi: 10.1371/journal.pone.0122700 (PMC4409205; doi:10.1371/journal.pone.0122700)

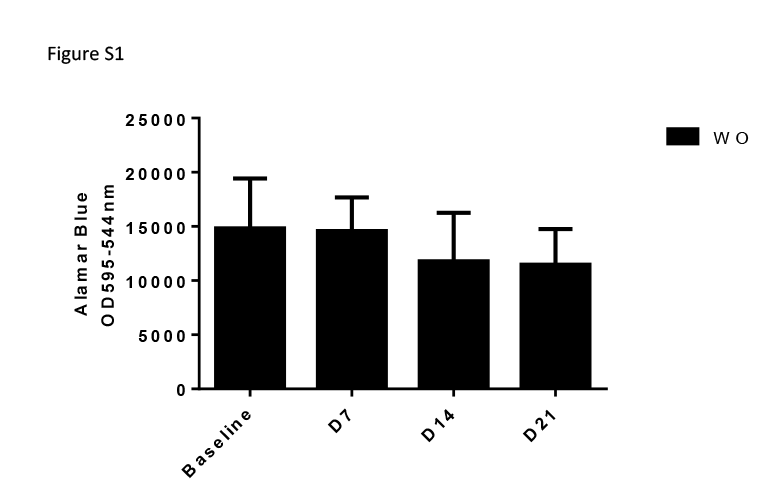

Supplement: S1 Fig — the cell viability of the harvested explants stabilized after 7 days in serum-free medium, but had a tendency to decrease gradually over time from 14823 (OD value) to 11461 at day21, although these changes were not statistically significant. (TIF) [file pone.0122700.s001.tif]

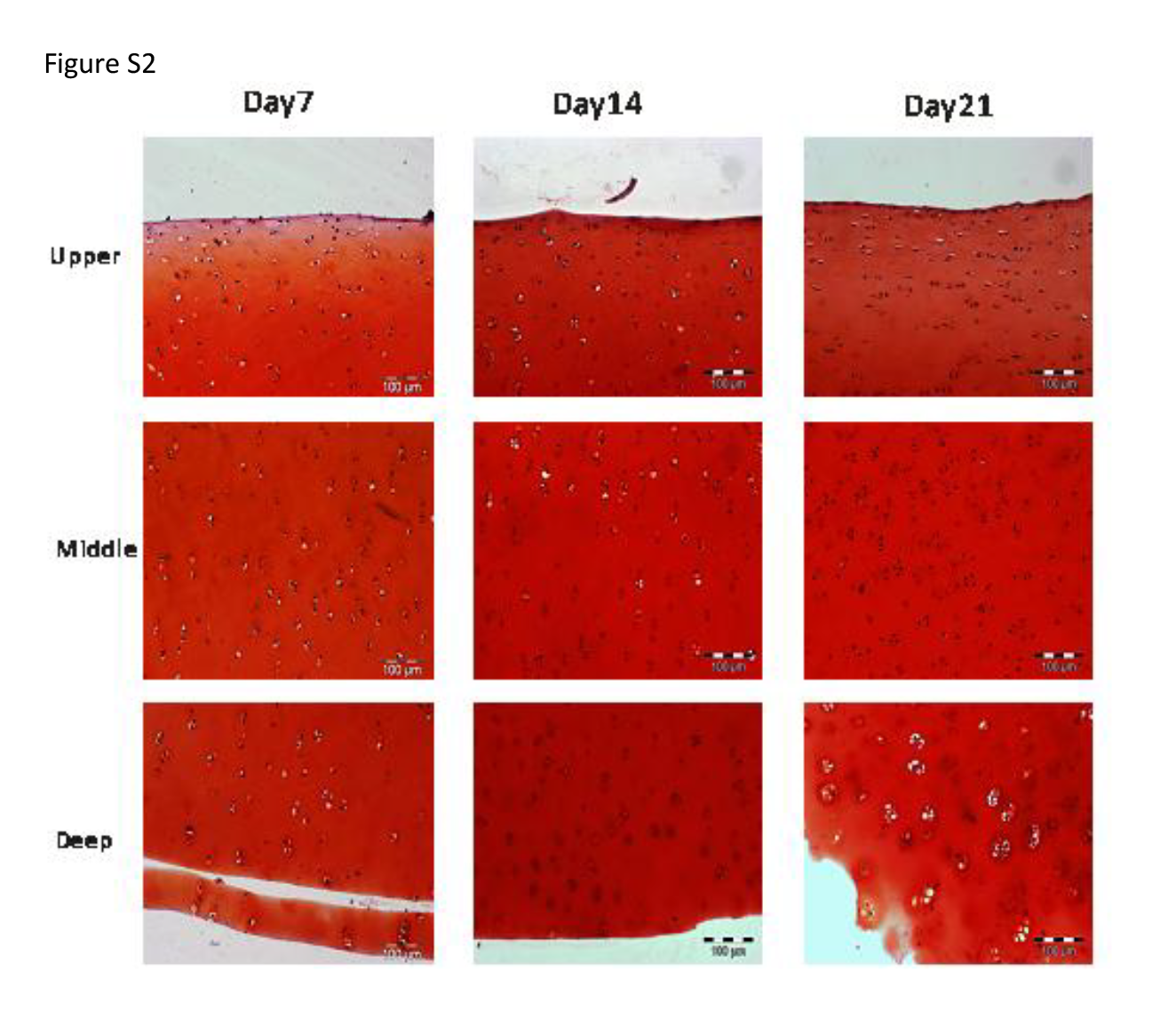

Supplement: S2 Fig — There was no significant loss of the proteoglycan of three zones (upper, middle and deep zones) after 21 days culture in serum-free medium (TIF) [file pone.0122700.s002.tif]

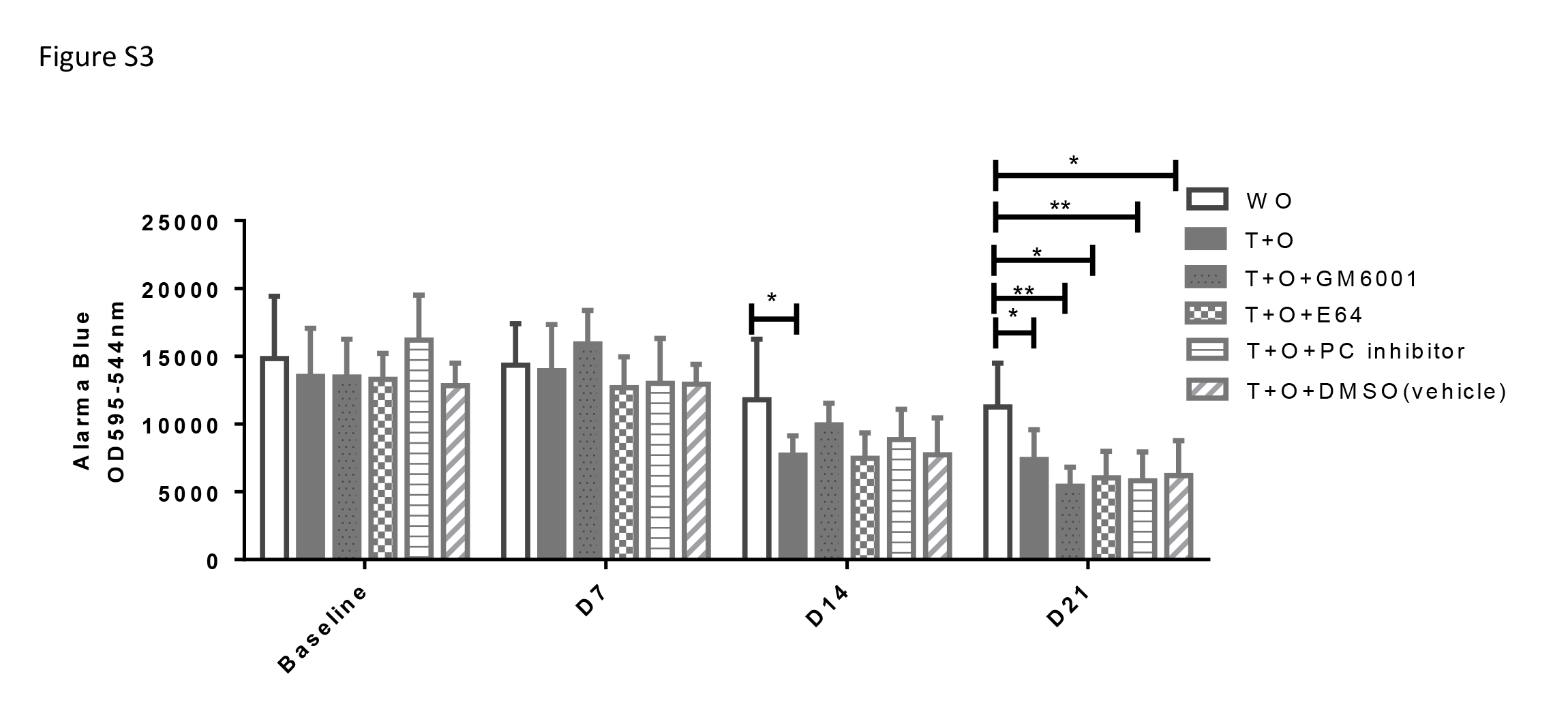

Supplement: S3 Fig — All values were shown as mean±95% confidence intervals (n = 8 in each group). Error bars indicated the upper limit of 95% confidence intervals. The cell viability of the harvested explants stabilized after 7 days culture in serum-free medium, and then decreased gradually over time, although these changes were not statistically significant. The cellular viability was not affected by stimulators, inhibitors or vehicle after 7 days until it dropped significantly at day 14 since there was cartilage degradation induced by stimulators. The addition of inhibitors improved the cell viability to some extent compared to T+O alone group. On day 21, the cell viabilities of T+O with or without inhibitors groups were significantly lower than the WO group, but no significant difference was detected among the groups. The data indicated that the distinct responses of biomarkers in each group resulted from the changes in the metabolism instead of the viability of chondrocytes caused by the inhibitors. (TIF) [file pone.0122700.s003.tif]
